# Physical activity and hybrid closed loop technology in adults with type 1 diabetes mellitus – what are the challenges experienced by the users and how are these met? A qualitative interview study

**DOI:** 10.1186/s12902-026-02297-9

**Published:** 2026-05-13

**Authors:** Frida Juliussen Hjortdahl, Eirik Årsand, Ragnar Martin Joakimsen

**Affiliations:** 1 Faculty of Health Sciences, University of Tromsø - The Arctic University of Norway, Tromsø, Norway; 2 Department of Computer science, University of Tromsø - The Arctic University of Norway, Tromsø, Norway; 3 Institute for Clinical Medicine, University of Tromsø - The Arctic University of Norway, Tromsø, Norway

**Keywords:** Type 1 diabetes, Physical activity, Exercise, Hybrid closed loop technology

## Abstract

**Objective:**

Type 1 diabetes mellitus (T1DM) requires lifelong insulin treatment based on blood glucose (BG) values affected by many factors, making the treatment complex. Automated insulin delivery through hybrid closed loop (HCL) technology has now shown to be an effective and safe treatment option. However, these systems still perform sub-optimally during physical activity (PA). This study aimed to analyze challenges experienced during PA by people with T1DM using HCL technology and how they addressed these challenges.

**Methods:**

We conducted a qualitative interview study based on semi-structured individual interviews with five participants. All participants used the Medtronic 780G insulin pump. Systematic text condensation was used as analysis method.

**Results:**

The main findings of the study are summarized in five major themes: (1) Challenges in Optimisation of Pre-Conditions for Exercise, (2) Limitations of user equipment, (3) Challenges in maintaining glycemic control during PA, (4) Challenging consequences after PA and (5) Need for manual override.

**Conclusion:**

Achieving good BG control during PA is challenging despite good prerequisites and use of HCL technology. There is a need for improvements in attaching the pump and sensor, more accessible ways to monitor sensor glucose values, and increased education. Specifically, more opportunities for regulating insulin delivery during PA, and better guidance that focuses on how to avoid both hypo- and hyperglycemia in relation to PA when using HCL technology.

## Introduction

Type 1 diabetes mellitus (T1DM) is a chronic autoimmune disease that requires lifelong insulin treatment to regulate the blood glucose (BG) [1]. BG levels and insulin requirements are affected by many factors, making the treatment complex [2]. The traditional insulin pump, CSII, requires a fixed program for a basal dose that is injected regularly throughout the day, and the patient must administer bolus doses before meals and when correction is needed. Today, more automated solutions have been developed for the treatment of T1DM, in the form of insulin pumps with hybrid closed loop (HCL) technology that are connected to continuous glucose monitors (CGM) [3, 4]. A CGM continuously measures glucose in the interstitial fluid via a subcutaneous sensor, with sensor glucose (SG) differing from BG, as it´s measured in interstitial fluid, not blood [5].

Insulin pumps with HCL technology uses algorithms to deliver insulin based on SG values and trends, active insulin in the circulation, insulin sensitivity, registered carbohydrates (CH), insulin-to-carb-ratio and other factors. They automatically adjust the basal dose, lessening the patient´s need for glucose monitoring and insulin assessment [6]. The aim of this treatment method is to increase the time in range (SG value between 3.9 and 10 mmol/l), reduce hypo- and hyperglycemia and reduce the burden of disease [7]. The term “hybrid” means that the system requires special management for factors such as meals and physical activity (PA) [8] – thus, it is not a fully automatic closed-loop system. The automatic function of HCL systems can be switched off, making the pump work as a standard CSII [8, 9]. This also applies to the insulin pump Medtronic 780G with Guardian 4 sensor [9], which all of the participants in this study used. This is the most commonly used insulin pump with HCL technology in Norway [10], where the study was conducted.

Meta-analyses show that HCL systems are an effective and safe treatment method for T1DM [11] and that they reduce the risk of severe hypoglycemia [12]. HCL technologies reduce the incidence of hypoglycemia during PA compared to other treatment methods for T1DM and have been shown to be safe to use during unplanned PA [13–15]. However, challenges remain, with PA still considered one of the greatest challenges [15, 16]. Since the HCL system calculates the insulin dosage based on a desired target glucose value (among other factors), it is recommended to increase this value 1–1.5 h before PA to reduce the amount of active insulin in the body during the activity and thus the risk of hypoglycemia. This function is called a temporary target (or exercise target), and is necessary for the HCL system to take into account that the user is performing PA.

SG values usually correlate well with BG when BG is stable [17, 18]. The faster the glucose value in the interstitial fluid changes, the greater the discrepancy will be [19, 20]. This is particularly a challenge in relation to PA, which can cause rapid changes in glucose values, as well as the delay in the BG value compared with the SG value. These factors imply that SG values in some contexts can deviate considerably from BG values, which constitutes a significant challenge for HCL systems, especially in relation to PA and CH intake [15, 21]. Consuming CH before or during PA is a strategy to reduce the likelihood of, or treat, hypoglycemia. Some choose not to inform the pump about CH intake in relation to PA to avoid receiving bolus doses of insulin. This is described as a further challenge for the HCL systems [15], since this leads to incorrect information in the pump, which the subsequent calculations will be based on. In addition, the CGM will register a rise in SG after CH intake and the insulin supply will increase [22].

PA is beneficial for people with T1DM in terms of insulin sensitivity, lipid profile, risk of cardiovascular disease and psychological endpoints, but it has not been shown to improve glycemic control [15, 16]. Maintaining work and leisure activities involving PA while living with T1DM is crucial for preserving quality of life and reducing the burden of the disease [23]. Individuals with T1DM perform less PA than control groups without T1DM [24], mainly due to fear of hypoglycemia, loss of glycemic control and limited knowledge about managing treatment around PA. HCL technology aims to reduce the treatment burden for T1DM patients, with user experience being an important topic [25, 26]. However, several studies highlight that HCL technology face challenges with PA regarding endpoints such as hypoglycemia and challenges for the system itself and the algorithms used [25, 27]. There is a lack of research-based knowledge on how the users experience these challenges, which is crucial for achieving satisfying treatment results and compliance [26]. Therefore, the aim of this study is to take a closer look at: (1) What are the challenges experienced in PA when using HCL technology in the treatment of T1DM and (2) How are these challenges met by the users?

## Materials and methods

We conducted a qualitative interview study with semi-structured individual interviews involving five participants. The interpretive paradigm, underpinning qualitative methods [28], has been used as a basis for understanding. A female medical student (FJH) conducted the study for a master thesis in medical professional studies at the University of Tromsø – The Arctic University of Norway (UiT), supported by two male supervisors: a professor in cyber-physical systems (EÅ, PhD) and an endocrinologist/professor (RMJ, PhD).

### Ethics

The Regional Committee for Medical and Health Research Ethics (REK) found that this project did not require an application under the Norwegian Health Research Act (ref 417336/REK Nord, S1 appendix). We received approval from the research responsible institution’s Data Protection Officer (S2 appendix, 2021-11-10) and the Norwegian Centre for Research Data (ref 962742/S3 appendix). Participants were informed in writing and verbally about the study and their rights prior to the interviews and provided written consent to participate in the study. Audio recordings were made with written consent (S4 appendix) and stored on an encrypted offline memory stick. The material was processed offline. Transcriptions were done by the interviewer (FJH) and the audio recordings were deleted within 7 days of post-interview. The study was carried out in accordance with the declaration of Helsinki. Clinical trial number: not applicable.

### Participants and recruitment

We aimed to recruit three to five participants through the local diabetes outpatient clinic at the University Hospital of North-Norway and social forums, with approval from the Norwegian Centre for Research Data (ref 962742/S3 appendix). Participants were recruited within the timeframe 2022-06-26–2023-01-22. Interested individuals contacted a researcher (FJH) by email. The inclusion criteria for participation in the study were (1) age over 18 years, (2) using an insulin pump with HCL technology for at least 8 weeks, and (3) regularly engaging in PA affecting BG, such as exercise or other activities, at least weekly for over 30 min per session. These criteria were aimed at capturing a wide range of activity-related experiences to gather rich and nuanced information on the relevant topic. All individuals who were interviewed were included in the study. We initially interviewed three participants and added two more to enhance information power.

### Data collection

Data collection involved the same researcher (FJH, a female medical student with no prior relationship to the participants) conducting an individual semi-structured interview with each participant, lasting 36–82 min (mean 41.2 min). Interviews were held at the University of Tromsø or via Microsoft Teams for remote participants. The “Critical Incident Technique” [28] inspired the interview questions, focusing on eliciting concrete event-based experiences. Questions were open-ended, exploring participants´ experiences with HCL technology during PA, challenges encountered and their responses. The interview guide (S5 appendix) was developed for this study by the three involved researchers (FJH, EÅ, RMJ) and refined after the initial two interviews to focus on emerging themes. The interview guide has not been published elsewhere. Participants were not compensated for their involvement in the study. No pilot interviews were conducted, and no repeat interviews were carried out.

### Data analysis

Data were analyzed using systematic text condensation (STC), a four-step method by K. Malterud [28], with Microsoft Word 2023 as the sole analysis tool. The interviewer and transcriber (FJH) performed the analysis, consulting with co-researchers (EÅ, RMJ). Initially, we reviewed all data for an overall impression and identified preliminary themes (step 1). Next, we extracted meaning units, coding them into themes (step 2). These units were then sorted into subthemes and condensed (step 3), followed by crafting an analytical text summarizing each subtheme´s essence (step 4). This process yielded five major themes with two to three subthemes each. We adhered to the COREQ guidelines for thorough reporting of qualitative research (S6 appendix).

## Results

The findings in this study are organized into the following major themes: (1) Challenges in Optimisation of Pre-Conditions for Exercise, (2) Limitations of user equipment, (3) Challenges in maintaining glycemic control during PA, (4) Challenging consequences after PA and (5) Need for manual override, summarized in Table 1. Characteristics of the five interviewees included are described in Table 2. All participants used the Medtronic MiniMed 780G insulin pump with Guardian 4 sensor. One participant had previous experience with Tandem Control-IQ. One participant had a low-carb diet. Activities performed by the interviewees were both low-intensive activities such as yoga, and more high-intensive activities such as running – see Table 2 for details. PA was performed for 30 min up to eight hours per day, either continuously or divided into multiple periods throughout the day. The frequency of PA performed varied from once a week to every day. The level of PA in the study population varied from those who engaged in PA on a hobby and necessity basis to those who engaged in more serious and targeted training.

**Table 1:** Main challenges associated with the identified themes

Themes → Challenges ↓	1: Challenges in Optimisation of Pre-Conditions for Exercise	2: Limitations of user equipment	3: Challenges in maintaining glycemic control during PA	4: Challenging consequences after PA	5: Need for manual override
	Little focus from health care personnel	Poor sensor attachment, reaction to tape	During long and intense activities	Elevated blood glucose levels	Periods with varying PA (less versus more PA)
	Lack of information	Tubing system hindering PAs	“Temporary target” function not sufficient	Need for switching to ‘manual’ mode	The pump needs time to adapt to new PA levels
	Lack of planning	Large, non-discreet system	System too slow to adapt to PA	Feeling that PA gave negative results in total	Lack of education in adjusting pump
	Missing meal information	Limited monitor-/adjustment options	Distrust to the ‘auto’ mode – chose ‘manual’	Feeling of mental and physical burden	‘Training days’ versus ‘resting days’
	Missing options for insulin adjustment	Beyond average technical skills required	How to correct hypo-/hyper-glycemia?	Require more time and effort than usual	Pump does not allow overriding insulin admin.

**Table 2:** Characteristics of the five interviewees

	Mean
Age, years	41 (SD = 11.4)
Duration of T1DM, years	22 (SD = 8.3)
Duration of treatment with an insulin pump with HCL technology, months	14 (SD = 7.3)
Hba1c, mmol/mol	53 (SD = 8)
	**Number**
**Gender**	
Male	1
Female	4
**Education**	
Bachelor’s degree or higher	5
Physical activities	Cycling, Strength training, Running, Yoga, Hiking and skiing, Swimming, Horseback riding, Domestic chores, and PA in a job context

### Theme 1: Challenges in optimisation of pre-conditions for exercise

**Insufficient focus on PA during instructions using the equipment**: Several participants felt that there was insufficient focus on PA during the instructions they received from the health care system prior to the use of the HCL system, and that this was not prioritized. This could contribute to fear of engaging in PA because they did not know how the pump worked in relation to activity, or how to handle problems that could arise. A lack of information on how to handle different types of PA, especially everyday activity, was also described. Insufficient knowledge of how to use the pump in relation to PA led some to have unmet expectations of the pump, reduced reliability, and lowered activity levels. One participant described it like this: *“If healthcare professionals who have training on these pumps have more focus on how you can perform physical activity […] in a good way […] then it won´t be so scary. […] In the beginning*,* you don´t trust such artificial intelligence. Getting more information can help you dare to be more physically active. And information that physical activity is not just about exercising. Because I think healthcare professionals who have the training forget about that.”*

**Challenging requirements for planning**: Several participants were advised to set temporary targets for a while before PA, which could be challenging due to planning and unforeseen activities. One participant reported that the pump likely would have worked better during PA if planning had been easier. The requirement to take precautions and try to plan was also described as limiting freedom. Planning PA in relation to meals could also be challenging, considering how long before activity one should eat, and how CH rich the meal should be. Several participants wanted more knowledge about such issues. Too much time between meals and PA could result in hypoglycemia, while too little time could cause the pump to correct rising SG during activity, leading to hypoglycemia. Consuming meals that were too CH rich before activity resulted in the pump delivering too much insulin, making it difficult to avoid hypoglycemia during the activity. The balance between optimal timing of PA in relation to meals was also described as challenging in connection with practical limitations in life. One interviewee described the issue with planning like this: *“[…] The problem with temporary target is that you must plan. When you´re visiting a place*,* you don´t plan to bike home in an hour*,* you leave when it suits you”.*

**More opportunities to influence basal insulin delivery during activity**: There were desires for greater opportunities to influence basal insulin delivery before or during PA. For example, with more choices for setting temporary targets. Existing options for setting temporary targets, as experienced by the participants, limited their ability to prevent hypo- or hyperglycemia during PA. Therefore, some participants chose not to use temporary targets. One participant described it like this: *“So I have given up on temporary targets*,* and actually*,* I would like to lower the target when I exercise. To ensure that I get enough insulin. I would like to have temporary target*,* but then I would have it in the opposite direction […]”.* It was also highlighted that this function could have been easier to find, for example, as one of the shortcuts on the pump.

### Theme 2: Limitations of user equipment

**Practical limitations**: Practical limitations with user equipment were highlighted as challenging in connection with PA. For example, with poor sensor attachment and rash due to the tape in relation to activity when one becomes sweaty. The fact that the pump has a tubing system was described as a cumbersome and logistical challenge, especially during running and swimming. It was described that the pump was often in the way during PA, that the tubing could get caught or fear that the pump would come loose and fall off. One participant described it like this: *“[…] You don’t necessarily want to*,* I mean*,* there´s a reason for why you have the pump*,* you don’t want to take it off every time you’re going to do something that makes it a nuisance either”.* Despite the fact that the pump is waterproof, the participants did not bring the pump into the water, which could make BG regulation during and after swimming challenging. The pump was also described as large and not very discreet under workout clothes. Possible attachment devices for the pump during PA were limited by the fact that it had to be accessible enough to monitor and possibly correct SG values.

**Need for increased accessibility**: Current limitation to monitor SG values only on the insulin pump was a challenge for several participants during PA. Therefore, many wished for the possibility to transfer SG values to other devices such as a smartwatch or -phone. One participant described it liked this: *“[…] For the manufacturer*,* it may sound strange that it´s so important to get the blood sugar somewhere else than on the pump*,* but when you go skiing in 20 degrees below zero*,* you can´t just take out the pump from under all the clothes*,* or have it in your peripheral vision*,* it just doesn’t work”.* Some participants had managed to transfer SG values to their smartphone or -watch (however only used as alarms for high and low values and not continuous value), while others had not. There is a mobile phone app that enables display of SG values from this pump, but this is only compatible with certain phone models. However, some participants were unable to transfer the SG values to their phone, despite having a compatible phone. Participants described that they were dependent on luck and technical background to make this work. The need to be able to do this in a standardized way was therefore emphasized by the interviewees.

### Theme 3: Challenges in maintaining glycemic control during PA

**Challenges with PA of different duration**,** intensity**,** and character**: Participants described different challenges with different types of PA, where duration and intensity were particularly important factors. It was particularly challenging to maintain desirable SG values during long, continuous activity – even though temporary targets were turned on well before the activity began. During high-intensity PA, several participants experienced greater challenges in the form of BG deviations than during lower intensity, and an increased need for self-monitoring (in terms of CH intake or administering bolus doses of insulin). Activities involving feelings of stress were also described as particularly challenging. One participant described it like this: *“If the duration is not so long and the intensity is not so high*,* then it´s possible to use temporary target. But if the duration is longer and the intensity is higher*,* then I must take off the pump to avoid getting low blood sugar”.* The type of activity and thus how practical it was to take a break to consume food or administer insulin, was reported to have a significant impact on BG regulation, safety, and how limited one became during PA.

**Too slow adaptation to different insulin needs**: In our material, several participants felt that the pump did not correct and adapt to different SG values quickly enough during PA in auto mode, resulting in large SG deviations. This was particularly challenging when activity intensity varied, leading some participants to use their pump in manual mode instead of auto mode. It was described that when exercising with the pump in auto mode, it allowed SG to deviate somewhat from the target value until a threshold was reached where the deviations accelerated, and the pump could not correct SG back to the target value for a long time. Participants who performed PA often suspected that this could have a great impact on HbA1c. Although all participants were aware that SG has a delay compared to actual BG, they had all made a conscious choice to rely on SG values. At the same time, they were painfully conscious that this meant more variation in BG than indicated by SG during rapid glucose fluctuations and deviations. Consequently, it was difficult daring to let go of the control during PA, and easier choosing to regulate SG in manual mode. One participant described it like this: *“I have tried to exercise with the pump in auto mode*,* but when I get this increase (in SG)*,* it is not quick enough to bring it back down. And then I get over 12 in sensor glucose*,* and when I then measure in my finger*,* it’s 15. So*,* then it´s inaccurate*,* because it´s a bit delayed […]. But I have done it (exercised with the pump in auto mode)*,* but I don’t do it anymore because it hasn’t worked well enough”.*

**Need for self-correction**: Most participants had to consume CH or administer insulin doses during PA to avoid unwanted SG deviations, and for some, this did not correspond to the expectations they had for the pump. Several interviewees found it challenging to find an optimal balance between energy intake and expenditure during activity with the pump in auto mode, as well as insulin dosing when the pump was not in auto mode. More knowledge was desired on correcting hypo-/hyperglycemia during PA when using HCL technology. Some participants had found a balance between necessary nutrient intake in relation to energy expenditure and insulin needs during activity. Although these also had to adjust BG manually during PA, they did not experience it as negative because the balance they achieved between insulin levels and BG was perceived as favorable for performance. These participants rarely experienced hypoglycemia during PA. One participant described it like this: *“But of course*,* if I had left all backup food and stuff at home*,* and just went to a training session without bringing anything*,* it wouldn’t have worked. But I don’t think we´ll ever get a system that can handle that. You must bring food with you… I mean you use a lot of energy when working out*,* so that´s maybe okay”*. Some participants had to consume CH or maintain higher SG values during activity for the pump to provide enough insulin, as they could not adjust basal insulin delivery in auto mode. If the pump suspended insulin delivery for too long during activity, feelings of high BG were described even though BG was normal, something they suspected to be due to insulin deficiency.

### Theme 4: Challenging consequences after PA

**Undesirable BG values after PA**: Several participants highlighted that elevated BG levels after PA were a challenge. Some participants experienced substantial BG deviations after PA that required switching to manual mode instead of remaining in auto mode (as during PA). The participants had been advised to leave the pump set to ‘temporary target’ for a while after activity, but several did not experience sufficient effect from this. Undesirable BG values after PA gave a negative association with PA, hampering the good feeling of taking care of one´s health by getting hypo- or hyperglycemia afterwards. One participant described it like this: *“It’s frustrating because you exercise for many reasons*,* to take care of the health among other things*,* and when I then end up with high blood sugars afterwards*,* I feel like I’m ruining it. It leaves a bit of a bad taste”.* Some of the participants who used their own solutions for BG regulation during PA described that this could contribute to challenges after PA. For example, if one took off the pump during activity and subsequently got hyperglycemia afterwards due to the lack of insulin delivery. One participant described that (s)he did not use temporary target during activity due to insufficient effect of this, as well as not informing the pump about CH intake during PA. These strategies worked well during the activity itself but posed challenges with hyperglycemia after activity.

**Psychological and somatic burdens**: Several participants experienced that engaging in PA using an insulin pump could be mentally and physically burdensome. The need to engage in correction of BG during and after PA resulted in some feeling like a burden if they engaged in PA in the company of others, and challenges with performing physical work in a job context. This could affect self-esteem and performance. Feelings of being punished for engaging in PA when BG regulation during and after activity was suboptimal were described. Several had often refrained from engaging in PA because it was easier to maintain satisfying BG regulation when they were inactive, and because they were tired of all the effort it took trying to have well-regulated BG. Setting aside time for breaks due to hypoglycemia, correction needs and recovering from BG deviations was highlighted as challenging in a busy life. Safety was also emphasized, with several describing that they trusted the pump more when they were not engaging in PA than when they were in PA. One participant summarized it like this: *“I don’t really notice that I have diabetes so much anymore after I got this pump*,* because it (BG) is regulated so well. But particularly exercising and physical activity are some of the things that still are very challenging”.*

### Theme 5: Need for manual override

**Challenges with periodic changes in activity level**: It was reported experiences where the HCL technology had challenges adapting BG regulation to periods of varying physical activity levels. For example, when transitioning from a period of less PA to a period of more PA and vice versa, such as with shift work involving physical labor, and vacations. When transitioning from a less to a more physically active lifestyle, several experienced that the pump did not detect this and based insulin dosing on a lower activity level than reality, which could result in multiple episodes of hypoglycemia daily. This was especially an issue during the first few days after a change in activity level. The participants who experienced this did not know how to handle the problem as they had not learned about it. When contacting the health care system for help, advice was given to turn off the auto mode and use manual mode until the pump had adapted to the new activity level. Being used to auto mode, that could be challenging. One participant described it like this: *“I had a longer period of inactivity at work (before I became more active)*,* so the pump was used to a much higher blood sugar and lower activity level. So*,* in the beginning (when I got a job with more physical activity)*,* I had to turn off auto mode because it just drove me down no matter what I did. I experienced that the pump took completely over […]*,* and I was low at least 3–4 times a day”.*

**Insufficient opportunities to override the HCL system**: Many participants found that the pump worked well if one lived a routine life and in normal situations, but they encountered challenges outside of these situations. For participants who exercised a lot, training days was the normal situation, and then the problem was that the pump did not handle rest days or sick days, because insulin needs changed compared to normal. In these situations, there was often a need to leave auto mode to regain BG control as the pump took too long to do this on its own. There was a desire to be able to override the pump when in need of more insulin than the pump allowed to administer. This also applied to meals after PA, when the pump did not allow the user to administer the desired amount of insulin due to suspended basal insulin delivery during low SG levels. If one had to eat a meal for various reasons anyway, it resulted in subsequent hyperglycemia and frustration not being able to reasonably override. One participant described it like this: *“The fact that I’m not allowed to say that now I need more insulin*,* I know it*,* I can take the responsibility*,* so yeah… if the pump had said “yes okay*,* you can do it*,* but then you have to take the responsibility yourself”*,* then I would have done it […]. Even if I had to sign a document saying I took responsibility. Just having the opportunity to contribute would have been a huge improvement. Then I’m absolutely sure that I would have had a lower HbA1c”.*

## Discussion

Our qualitative study aimed to study challenges experienced by the insulin pump users during PA when using HCL technology, as well as how these challenges were handled. Based on the results above, the findings and their possible implications are discussed in relation to three perspectives: “timing”, “practical challenges” and “knowledge and strategies”.

### Timing

Our study´s findings on challenges with planning and unexpected activity are tied to insulin pharmacokinetics and the necessity to inform the pump about upcoming activity, which is supported by existing literature as one of the main challenges with HCL technology and PA [15, 25, 29]. The user must inform the pump that (s)he will perform PA by turning on temporary BG target 1–2 h before PA, so that the amount of active insulin in the circulation has time to fall before activity starts [9, 15, 29]. However, for several participants in our study, temporary target did not have sufficient effect even though it was turned on well in advance of PA. It was therefore desirable to have the ability to set the value higher or lower than what is currently possible. The challenges participants experienced with the pump not adapting to different insulin needs during PA quickly enough are also likely related to insulin pharmacokinetics. Sensor delays can also contribute to this. However, several participants experienced that they could handle BG regulation in manual mode during activity better than the pump could in auto mode, despite the users still having to take into account insulin pharmacokinetics and delays in sensor values.

### Practical challenges

In another qualitative study on patient´s prospective expectations of using HCL technology, patients were concerned about the practical limitations of the pump in relation to PA [30]. Our participants also found challenges with attaching the (relatively large) pump to the body during PA, tubing system, and sensor attachment. Practical limitations in relation to user equipment have been described in the literature as a possible challenge for the further development of HCL technology regarding the inclusion of, for example, heart rate monitoring to make BG regulation more precise during PA [27, 31]. SG monitoring was highlighted as a significant practical challenge in this study, with several participants believing it to be of great help if there had been opportunities to see SG not only on the pump.

### Knowledge and strategies

#### Receiving instructions of use

It is known that people with T1DM engage in less PA than people without T1DM. Insufficient knowledge about how to manage BG regulation during PA plays a central role [24, 29, 32]. Our study confirms that this applies even when using HCL technology, especially in case of inadequate instructions of use. Several participants wanted more knowledge about how to use the pump in relation to PA and believed that this would have contributed to increased confidence and performance of activity. However, existing guidelines for safe performance of PA in T1DM primarily focus on patients treated with MDI or CSII and/or CGM. HCL-systems are still relatively new, and there is limited available information regarding their use in relation to PA [15]. This requires approaches that are not yet standardized [15, 16, 29].

#### Carbohydrates

In our results, it emerged that planning and calculating CH intake in relation to PA was a challenge for which several participants had found no good solution or strategy. There is a lack of studies on recommended amounts and optimal timing of CH intake in relation to PA, making it difficult to provide precise advice to the patients, and to make individual adaptions [27]. Our study shows that it can be difficult for patients to find such solutions themselves. One participant in our study who had a low carb diet described fewer challenges with BG regulation in connection with PA than several of the other participants. Another participant who had found a strategy for CH intake versus consumption during PA also had fewer challenges during activity than participants who had not. Timing and intake of CH amounts in relation to PA while using HCL technology differs from such conditions when using CSII or MDI because insulin delivery is automated [15]. Findings from our study support the need for tailored strategies for CH intake in connection with PA when using HCL technology. In some existing literature, it is described that one should distinguish between CH intake in connection with PA that is necessary to avoid hypoglycemia and CH intake that is necessary for performance. Most studies have investigated this in relation to avoiding hypoglycemia [29]. Some participants in our study described that they had to eat CH during PA so that the pump would deliver insulin if it had suspended insulin delivery for a while to avoid hypoglycemia. If the participants did not do this, they described getting symptoms of hyperglycemia despite normal glucose values on BG/SG measurement, which they interpreted to be due to insulin deficiency (because of the pumps insulin suspension over time). The feeling of insulin deficiency experienced by the participants was also described to affect performance. This may be particularly challenging with the use of HCL technology because insulin delivery is automated, and thus the user has less control over it than with MDI or CSII [29].

#### Hyperglycemia after activity

A substantial portion of the existing literature in the field focuses on strategies to avoid hypoglycemia after PA, especially nocturnal hypoglycemia [15, 27, 29]. In this study, few participants had challenges with hypoglycemia after activity, while many had challenges with hyperglycemia. This can be interpreted as a result of how the pump was used during activity and/or in combination with strategies to avoid hypoglycemia. Participants who removed the pump during activity experienced hyperglycemia after activity due to a lack of insulin delivery. Those who consumed CH during activity without informing the pump also experienced hyperglycemia after activity. This strategy is understudied with HCL technology [15], and our results suggest that it presents a challenge.

#### Variations and manual override

Several participants in our study had developed their own strategies for managing BG regulation during PA when the pump did not do this adequately. In another study on patients’ experiences with the use of HCL technology, it was found that people with HbA1c below 7.5% more often felt the need to override the pump than people with higher HbA1c. These individuals more often felt that the pump did not meet their standards for glycemic control [33]. In our study, there was an expressed need for opportunities to perform manual override especially in relation to deviations from normal situations and in situations where the user meant they knew better than the pump which necessary actions should be taken in relation to insulin dosing. The participants in question tried to give the pump time (days to weeks) to adapt to different insulin needs without it succeeding, and it could also apply to situations with short-term changes in insulin needs (hours to days). In such situations, it can be reasoned that giving the pump long time to adapt can be challenging. Being able to override the pump in these situations was described as necessary to avoid the pump taking too much control with serious BG deviations as a result.

### Strengths and weaknesses

The qualitative approach in this study contributed to a richer and more nuanced material than if we had used a quantitative approach, because the questions would then have been predetermined not giving space for nuances and unexpected topics. The study had a relatively low number of participants with its five interviewees, which may limit the external validity of the study. Based on the sample’s comprehensive and various engagement with PA, it was considered sufficient to illuminate the research question in a satisfactory manner. All participants used the same type of insulin pump and sensor (Medtronic MiniMed 780G with Guardian 4 sensor), which may be thought to weaken the information breadth in the material and should be taken into consideration regarding the generalizability of the findings. However, it provided higher information power for this specific pump system (still the preferred HCL system by Norwegian health authorities per March 2026). All participants had higher education and relatively well-regulated BG based on HbA1c levels, which is likely relevant for the generalizability of the findings. The participants who chose to participate in the study may have been individuals who have reflected more on the topic of the research question and experienced challenges to a greater extent than participants who did not choose to participate. This may have contributed to selection bias, while also providing higher information power in the sample.

## Conclusion

Based on our results we conclude that there are still considerable challenges associated with the use of HCL technology in relation to PA that weigh negatively in terms of disease burden, even among individuals with favorable conditions for success with BG regulation in general. These challenges include challenges in optimisation of pre-conditions for exercise for use of HCL technology in relation to PA (in terms of insufficient education, requirements for planning, and lack of opportunities to influence basal insulin delivery during PA), limitations of the user equipment, difficulties in maintaining glycemic control during PA, challenging consequences after PA and the need for manual override. According to user experiences, there is a need for better practical solutions for attaching the pump and sensor, as well as more accessible ways to monitor SG values. There is a need for increased education regarding the use of HCL technology and PA. Better strategies for carbohydrate intake in relation to PA and the use of HCL technology, as well as for manual override, should be more focused. Specifically, more opportunities for regulating insulin delivery during activity are desirable from a user perspective. Thus, there is a need for more and better guidance that focuses on how to avoid both hypo- and hyperglycemia in relation to PA when using HCL technology.

## Data Availability

The data that supports the findings of this study consists of transcriptions from individual interviews. This data cannot be shared openly to protect study participant privacy. The data can only be shared with the publishing journal and no other researchers, due to restrictions from the Norwegian Centre for Research Data (Sikt).
